# Supplementary material for: Development of the Tool for Advancing Practice Performance, a practice-level survey to assess primary care structures and processes
Source: PLoS One. 2025 Sep 17;20(9):e0315558. doi: 10.1371/journal.pone.0315558 (PMC12443247; doi:10.1371/journal.pone.0315558)
Supplement: S1 File — (PDF) [file pone.0315558.s001.pdf]

S1 File. The Tool for Advancing Practice Performance (TAPP)

An online interactive version of the TAPP is also available at https://tapphealth.org/.

The first set of questions is about access to care at this practice.

1. Please indicate which of the following are true of your practice:

	Yes	No	Don't Know/ Unsure
a. PCPs (MD, DO, NP, PA) are available outside of the practice's regular office hours to respond to patients who call			
b. Practice is open outside of typical weekday hours (e.g., 9-5)			
c. Practice offers same day and/or walk-in appointments			
d. Practice offers group visits/shared medical appointments with multiple patients			
e. PCPs conduct in-home visits			
f. Staff other than PCPs (e.g., nurses, social workers, community health workers) conduct in-home visits			
g. Practice offers point-of-care testing (i.e., onsite) for certain lab tests			
h. Practice offers scheduled multidisciplinary visits onsite (or virtual) for patients (e.g., PCP and specialist meet jointly with patient; PCP and case manager meet jointly with patient)			
i. Practice offers option for longer **scheduled** appointments based on patient need			
j. Practice uses a standardized telephone triage assessment to inform patient if and where to seek care			

k. Patients have online access to their medical records			

The next section explores the various ways this practice communicates with patients.

2. Please indicate which of the following your practice uses to communicate with or monitor patients:

	Yes	No	Don't Know/ Unsure
a. Patient portal to track self-management goals			
b. Patient portal to communicate with patients			
c. Bidirectional communication with patients via text or email			

The following set of questions identifies any screens routinely utilized by the practice.

3. Please indicate for which of the following the practice uses formal tools to **routinely** screen all patients:

	Yes	No	Don't Know/ Unsure
a. Alcohol use			
b. Drug use			
c. Tobacco use			
d. Mental health symptoms			
e. Health literacy			
f. Social needs or social determinants of health (e.g., food insecurity, legal problems, at-risk for homelessness)			

The next set of questions is about patients' experiences with care.

4. In this practice, how often do patients see the same PCP (other than vacation time)?

- ○ Never/Rarely
- ○ Sometimes

- ○ Usually
- ○ Always
- ○ Not Applicable/Solo PCP

5. Does the practice offer to include family members in shared decision-making conversations?
   - ○ Yes
   - ○ No
   - ○ Don’t Know/Unsure

6. Does the practice have onsite PCPs or staff that speak the most dominant, non-English language spoken by patients?
   - ○ Yes
   - ○ No
   - ○ Don’t Know/Unsure

The following question is about this practice’s requirements for formal training.

7. Please indicate which of the following are required formal trainings for providers:

	Yes	No	Don’t Know/ Unsure
a. Cultural competency			
b. Implicit bias			
c. Motivational interviewing			

The next section is about this practice’s care team(s) including its/their makeup and function.

8. Does this practice divide patients into panels that are assigned to specific PCPs and/or care teams?
   - ○ Yes
   - ○ No
   - ○ Don’t Know/Unsure
   - ○ Not Applicable/Solo PCP

9. Does this practice have designated staff to manage patient panels?

- ○ Yes, medical assistants act as panel managers
- ○ Yes, PCPs act as panel managers
- ○ Yes, other staff act as panel managers
- ○ No, no staff dedicated to panel management
- ○ Not Applicable, practice does not maintain patient panels

10. Please indicate the types of people who collaborate to provide care for patients in this practice other than PCPs. Note: These individuals do not need to be onsite or employed by the primary care practice.

	Yes	No	Don't Know/ Unsure
a. Medical assistant			
b. Clinicians other than PCPs (e.g., RN, LPN)			
c. Trained health coach			
d. Paraprofessionals/CHWs			
e. Care coordinator			
f. Nutritionist/dietitian			
g. Pharmacist			
h. Behavioral/mental health provider			

11. Does this practice facilitate regular communication between PCPs and care coordinators (e.g., regularly scheduled meetings, communication template)?

- ○ Yes
- ○ No
- ○ Don't Know/Unsure
- ○ Not Applicable, practice does not have care coordinators

12. Please read through each item and indicate your response:

	Yes	No

a. Practice regularly reviews roles and responsibilities with all PCPs and staff		
b. Medical assistants in this practice are responsible for tasks other than taking vitals (e.g., providing patient education; conducting screens, etc.)		

The following set of questions elicit any non-clinical patient services that this practice offers.

13. When needed, does this practice **routinely**:

	Yes	No	Don't Know/ Unsure
a. Assist patients with insurance-related issues (e.g., enrollment, getting approvals for procedures)			
b. Help patients determine benefit eligibility			
c. Provide support to patients on where to access lower-cost medications			
d. Refer patients to community resources outside of the office based on a social needs screening			
e. Arrange free or low-cost transportation services to and from the clinic			
f. Make referral appointments for patients			
g. Refer at-risk patients to staff for lifestyle promotion (e.g., exercise, diet)			
h. Help vulnerable patients navigate the health care system (e.g., make appointments for lab and radiology tests)			

The next section concerns how this practice engages with patients during visits.

14. Does this practice:

	Yes	No	Don't Know/ Unsure
a. Direct PCPs to assess and review patient goals pre-visit			

b. Provide all patients ages 40-79 with a cardiovascular disease (CVD) risk score during their visit			
c. Provide patients with clinical summaries after each of their visits			

The next set of questions concerns care coordination (within the practice and externally).

15. Please indicate whether this practice **routinely**:

	Yes	No	Don't Know/ Unsure
a. Identifies and contacts patients with chronic illnesses about unmet chronic care needs (e.g., poorly controlled diabetes or blood pressure)			
b. Reminds patients of upcoming appointments			
c. Follows up with patients who have cancelled or missed appointments			
d. Uses patient-initiated contacts (e.g., medication refill request) to reassess patients' other needs (e.g., need for follow-up appointment)			
e. Schedules follow-up appointments before patients leave office			
f. Identifies and contacts patients about preventive care needs (e.g., vaccines and screenings)			
g. Generates lists of patients by specific conditions (e.g., patient registry)			
h. Monitors high-risk populations with registries or other systematic methods			
i. Provides clinical care management for high-risk patients			
j. Uses pre-visit planning data to identify patients to meet with care managers			
k. Performs medication reconciliation			

l. Allows someone in the practice who is not the patient's PCP to change medications			

16. Does this practice:

	Yes	No	Don't Know/ Unsure
a. Contract with external organizations to provide comprehensive care management for medically complex patients			
b. Contract with a behavioral health organization			
c. Facilitate direct communication between PCPs and specialists to whom they routinely refer (e.g., shared EHRs, co-location)			
d. Facilitate bidirectional communication between PCPs and mental health specialists (e.g., shared EHRs, co-location)			
e. Have a system in place to receive clinical information (i.e., assessment and treatment plan) from specialty referrals			
f. Refer patients to in-house specialists (e.g., cardiologists)			
g. Schedule routine visits with a pharmacist for patients			
h. Have processes in place to effectively manage care transitions to and from hospital and emergency department (e.g., practice uses protocols for scheduling timely visit after hospital/emergency department discharge)			
i. Work with inpatient case managers to identify high-risk patients who need follow-up support			
j. Use the EHR to view patient visits to other clinicians (e.g., specialists at other institutions/practices)			

The following section queries this practice's clinical information systems.

17. Which of the following does the practice have in place?

	Yes	No	Don't Know/ Unsure
a. Template/customized order sets for frequently used physician orders			
b. EHR-based clinical decision tool has built-in alerts for drug interactions			
c. Clinical decision support tool that allows PCPs to directly order diagnostic tests, medication, and counseling			
d. Reminders or best-practice alerts for services that a patient should receive			
e. A single EHR alert that includes all tests for which the patient was eligible at the time of visit and automatically linked to a preapproved order set			
f. An online or automated call-in system where patients can renew medications			
g. Computer-based order entry for prescriptions, procedures, or lab work			
h. EHR-generated alerts for non-formulary medication choices			
i. Social history template in patient encounters (e.g., living situation, family configuration)			
j. Structured templates for chronic disease management			
k. Requirement for PCPs to routinely update problem list in EHR			
l. Requirement for PCPs to assess and track medication adherence in EHR			
m. Standardized order sets			

The next set of questions concerns this practice's current experiences with telemedicine and remote monitoring.

18. Please indicate which of the following are true of your practice:

	Yes	No	Don't Know/ Unsure
a. Practice offers synchronous home-based telemedicine visits (e.g., video or telephone) with patients			
b. Practice provides patients with equipment (e.g., tablet) to use telemedicine from home			
c. Remote monitoring of patients by PCPs (e.g., on-going monitoring of patient data, such as blood pressure, blood glucose, weight)			
d. Practice combines home-based monitoring (e.g., blood pressure, blood glucose) with nurse-administered behavioral interventions			
e. Practice equips patients with appropriate self-management tools (e.g., glucometers, blood pressure monitors)			
f. Practice provides or facilitates access to virtual interactive modules for patients to learn problem-solving, self-management and/or related skills			
g. Practice provides or facilitates access to online tools or devices for patients to track self-management goals			

The following set of questions concerns the types of ancillary clinical services that are provided onsite at this practice.

19. Please indicate which of the following ancillary services are available onsite (i.e., not provided by a PCP):

	Yes	No	Don't Know/ Unsure
a. Nutrition counseling/diabetes education			
b. Mental/behavioral health services			
c. Social work services			

d. Health coaching			
e. Dental services			

The next section is about the types of meetings that this practice holds.

20. Which of the following meetings does your practice **routinely** have?

	Yes	No	Don't Know/ Unsure
a. Meetings between PCPs and staff to discuss patients and their care			
b. Position/role specific meetings (e.g., all MAs, all RNs, all administrators, all providers)			
c. Daily in-person or virtual "huddle" meetings			
d. All-staff meetings			

The following set of questions is about this practice's quality improvement activities.

21. Please indicate which of the following are **routine** activities in your practice:

	Yes	No	Don't Know/ Unsure
a. Set performance goals for the practice			
b. Track quality of care for the practice against performance goals			
c. Provide PCPs with individual performance feedback specific to their patient panel			
d. Build quality improvement activities into practice operations			
e. Dedicate time to reflect and evaluate following a practice change project (e.g., identify lessons learned, debrief)			
f. Apply process improvement methodologies (e.g., Lean principles, plan-do-study-act cycles)			

g. Provide protected time and resources to dedicated quality improvement team			
h. Have a system in place for data quality assurance (e.g., identify inaccuracies in their data)			
i. Use a visual dashboard that displays clinical quality measures to manage patients with chronic disease			
j. Use a visual dashboard that displays clinical quality measures to manage preventive care for patients			
k. Customize quality reports by patient characteristics (e.g., demographics, diagnosis)			
l. Actively track new clinical care guidelines and incorporate them into the practice			
